# Incidental pulmonary embolism in patients with cancer: prevalence, underdiagnosis and evaluation of an AI algorithm for automatic detection of pulmonary embolism

**DOI:** 10.1007/s00330-022-09071-0

**Published:** 2022-08-25

**Authors:** Peder Wiklund, Koshiar Medson, Johan Elf

**Affiliations:** 1 Department of Radiology, Region Halland, Lasarettsvägen 6, 30233 Halmstad, Sweden; 2 Department of Radiology and Functional Imaging, Karolinska University Hospital, Stockholm, Sweden; 3 Department of Physiology and Pharmacology, Karolinska Institutet, Solna, Sweden; 4 Department of Haematology, Oncology and Radiation Physics, Lund University, Skåne University Hospital, Lund, Sweden

**Keywords:** Pulmonary embolism, Venous thromboembolism, Neoplasms, Retrospective studies, Artificial intelligence

## Abstract

**Objectives:**

To assess the prevalence of reported and unreported incidental pulmonary embolism (iPE) in patients with cancer, and to evaluate an artificial intelligence (AI) algorithm for automatic detection of iPE.

**Methods:**

Retrospective cohort study on patients with cancer with an elective CT study including the chest between 2018-07-01 and 2019-06-30. All study reports and images were reviewed to identify reported and unreported iPE and were processed by the AI algorithm.

**Results:**

One thousand sixty-nine patients (1892 studies) were included. Per study, iPE was present in 75 studies (4.0%), of which 16 (21.3%) were reported. Unreported iPE had a significantly lower number of involved vessels compared to reported iPE, with a median of 2 (interquartile range, IQR, 1–4) versus 5 (IQR 3–9.75), *p* < 0.001. There were no significant differences in age, cancer type, or attenuation of the main pulmonary artery. The AI algorithm correctly identified 68 of 75 iPE, with 3 false positives (sensitivity 90.7%, specificity 99.8%, PPV 95.6%, NPV 99.6%). False negatives occurred in cases with 1–3 involved vessels. Of the unreported iPE, 32/59 (54.2%) were proximal to the subsegmental arteries.

**Conclusion:**

In patients with cancer, the prevalence of iPE was 4.0%, of which only 21% were reported. Greater than 50% of unreported iPE were proximal to the subsegmental arteries. The AI algorithm had a very high sensitivity and specificity with only three false positives, with the potential to increase the detection rate of iPE.

**Key Points:**

• *In a retrospective single-center study on patients with cancer, unreported iPE were common, with the majority lying proximal to the subsegmental arteries*.

• *The evaluated AI algorithm had a very high sensitivity and specificity, so has the potential to increase the detection rate of iPE*.

## Introduction

Venous thromboembolism (VTE), which includes pulmonary embolism (PE) and deep venous thrombosis (DVT), is a common complication in patients with cancer and is an important cause of morbidity and mortality. A significant number of all diagnosed PE are unsuspected events detected in computed tomography (CT) scans performed for staging or treatment response evaluation for example [1, 2]. The American Society of Clinical Oncology guidelines recommend that incidental PE (iPE) be treated in the same manner as symptomatic PE, and that treatment for isolated subsegmental PE should be offered on a case-by-case basis [3]. Prevalence of iPE ranges from 0.7 to 15% in different study populations [4]. This variability probably reflects the heterogeneity of included cancer types and stages, CT slice thickness, and single versus double read. Motion artifacts and suboptimal opacification of the pulmonary arteries may also lead to misdiagnosis of iPE. In addition, if iPE are identified based only on the radiological report or coding in the electronic health records, the iPE prevalence is likely underestimated, as unreported iPE are common. Previous studies using 1-, 4-, or 16-slice CT have shown that between 32 and 75% of iPE were unreported [5–7], while Bach et al, using 64-slice CT scanners, showed a 3.9% prevalence of iPE in cancer patients, of which almost 60% were unreported [8].

Methods based on artificial intelligence (AI) could be a way to increase the detection rate of iPE. Earlier studies have shown high accuracy for the detection of suspected PE in CTPA studies, with a sensitivity of 92.6–92.7% and specificity of 95.5–95.8% in retrospective studies [9, 10], and a sensitivity of 79.6% and specificity of 95% in a prospective study [11]. However, studies regarding iPE are lacking. The detection of iPE in standard chest CT scans is a different challenge, since the mean embolus burden is lower in iPE compared to suspected PE [1], while the scans are not optimized for visualizing the pulmonary arteries regarding both the risk for motion artifacts and inadequate pulmonary arterial contrast enhancement.

Thus, the objectives of the current study were to assess the prevalence of reported and unreported iPE in patients with cancer with regard to iPE level and multiplicity, and to evaluate a deep learning–based AI algorithm for detection of iPE in standard CT studies including the chest.

## Material and methods

### Design

This was a retrospective single-center cohort study conducted in Halland Hospital Halmstad, Region Halland, Sweden. The study protocol was approved by the Swedish Ethical Review Authority. Informed consent was waived because of the retrospective nature of the study.

### Patients

Patients were identified by screening all CT requests during the study period in the Radiology Information System. Inclusion criteria were patients of at least 18 years old with a confirmed diagnosis of cancer, at least one contrast-enhanced CT study including the chest, and an indication for the study other than suspicion of PE or follow-up after reported PE, during the period from July 1, 2018, to June 30, 2019. Age, sex, cancer type, and stage were recorded. Cancer types were grouped as following: very high (stomach, pancreas), high (lung, lymphoma, gynecologic, bladder, testicular), or low risk of VTE [12]. For all included studies, radiology reports were scrutinized for reported iPE.

### CT scan parameters

All studies were performed on a 64-slice multidetector CT scanner (Revolution CT, GE Healthcare), at 120 kVp, with mAs automatically chosen using automatic tube current modulation. Detector collimation was 64 × 0.625 mm, with a pitch of ~ 1. Contrast media volume and injection rate were chosen using the Omnivis calculator (GE Healthcare). Contrast media volume and flow rate varied depending on patient characteristics and type of scan, with 375 mg iodine/kg for a chest CT and 500 mg iodine/kg for a combined CT of the chest and abdomen, up to a maximum dose corresponding to a body weight of 80 kg. For example, a 70-year-old male weighing 80 kg with normal renal function would receive 86 mL contrast media (Omnipaque 350 mg/mL) at a flow rate of 2.9 mL/s for a chest CT and 114 mL at a flow rate of 3.8 mL/s for a combined CT scan of the chest and abdomen. Bolus tracking with monitoring of the attenuation in the descending aorta was used for all scans. For the combined protocols, dual acquisition was used. The scans were triggered when attenuation in the descending aorta reached greater than 100 HU, with a fixed delay of 18 s for the chest scan and 63 s for the abdominal scan. In cases of an estimated glomerular filtration rate (eGFR) < 45 mL/min, a reduced contrast media volume was considered on a case-by-case basis after reviewing the indication of the exam.

### Image review

A retrospective review of all included CT studies was conducted by the first author who is a general radiologist with 9 years of experience. All images were read in the picture archiving and communication system (PACS; Sectra, Sectra AB). iPE was diagnosed if there was an intraluminal filling defect in one or more pulmonary arteries that was not attributable to motion or flow artifacts for example, or to inadequate contrast opacification of the arterial tree. The axial 0.625-mm slice thickness images were used for the retrospective review, with reconstruction in the coronal and sagittal plane as needed.

All studies with an iPE were also reviewed by the second author, a radiologist with 6 years of experience including 1 year of subspeciality training in thoracic radiology. iPE cases were separated into four groups: single subsegmental, multiple subsegmental, segmental, and lobar or more proximal iPE, with final characterization of the iPE by consensus. In addition, the number of involved segmental and/or subsegmental vessels was estimated, where an embolus in a segmental or subsegmental artery was counted as one, while more proximal emboli were counted as the number of distally arising segmental arteries according to Qanadli et al [13], but without weighting for degree of obstruction. As the assumption was that smaller iPE in a single vessel or few vessels were more likely to be unreported, the number of vessels for each iPE case was emphasized over the arterial obstruction index.

Image quality of all studies was evaluated by the first author by measuring the attenuation of the main pulmonary artery. For the subjective evaluation, the most distal level that could be evaluated for iPE was recorded (central, lobar, segmental, or subsegmental arteries), taking into account pulmonary artery opacification, motion artifacts, streak artifacts, and image noise among other factors. In addition, overall image quality was scored based on the most distal level that could be evaluated: (1) good to excellent quality, allowing good to excellent confidence in verifying/excluding the presence of a PE; or (2) moderate quality, allowing moderate confidence in verifying/excluding the presence of a PE.

### Artificial intelligence (AI) algorithm

All CT studies that were included were analyzed by a commercially available deep learning cloud–based AI algorithm for PE detection and triage (Aidoc BriefCase, Aidoc Medical). In brief, the AI algorithm is a convolutional neural network trained and validated on tens of thousands of CT examinations acquired on a diverse range of CT scanners from multiple medical centers around the world. All CT studies were pseudonymized, transferred via secure upload, then analyzed by the AI algorithm. In the AI workflow, the AI algorithm uses an image recognition subroutine that looks at the actual images to verify that the images are compatible for analysis (for example, correct anatomical coverage, and if the study is a contrast-enhanced CT, CECT, or non-enhanced CT, NECT), and selects which series are to be analyzed.

All CT studies were reviewed first by the first author before being analyzed by the AI algorithm. Cases with a negative first review but positive AI result (suspicion of PE) were again reviewed by the first author.

### Statistics

Standard descriptive statistics were used on a per study level to compare cases with unreported versus reported iPE, and on a per patient level, to compare patients without iPE, with unreported iPE, and with reported iPE. Analysis of variance (ANOVA) was used to compare age and BMI between the three groups, with the chi-square test used for categorical variables. The Mann-Whitney *U* test was used for comparing the median number of involved vessels between unreported and reported iPE. Categorical data are presented as a percentage and continuous data as either the mean ± 1 standard deviation (SD) or the median with interquartile range (IQR). AI accuracy parameters are presented on a per study level. SPSS Statistics for Windows version 27 (IBM Corp.) was used for all analyses.

## Results

**Fig. 1:**
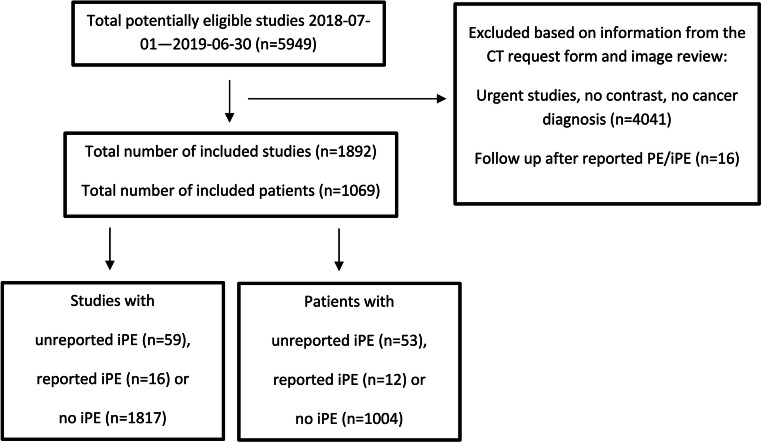
Flowchart of the inclusion process. PE, pulmonary embolism; iPE, incidental PE

A total of 1069 patients were included, having undergone 1892 elective CT scans during the study time period. The inclusion/exclusion process is visualized in Fig. 1. Image quality was graded as good to excellent to the subsegmental arteries in 68.8% of all studies, and of moderate quality to the subsegmental arteries in 16.5%, while in 14.7% the subsegmental arteries could not be evaluated. Image quality stratified for attenuation of the pulmonary trunk is presented in Table 1.

**Table 1:** Subjective image quality evaluation. The most distal level that could be evaluated for iPE is presented, stratified for attenuation of the main pulmonary artery. *HU PA*, HU of the main pulmonary artery

		Subsegmental, good-excellent	Subsegmental, moderate	Segmental	Lobar or more proximal	
HU PA	≥ 250	631	46	21	1	699
	200–250	325	26	13	5	369
	150–200	297	107	46	6	456
	100–150	48	133	145	13	339
	< 100	0	0	14	15	29
		1301	312	239	40	1892

**Fig. 2:**
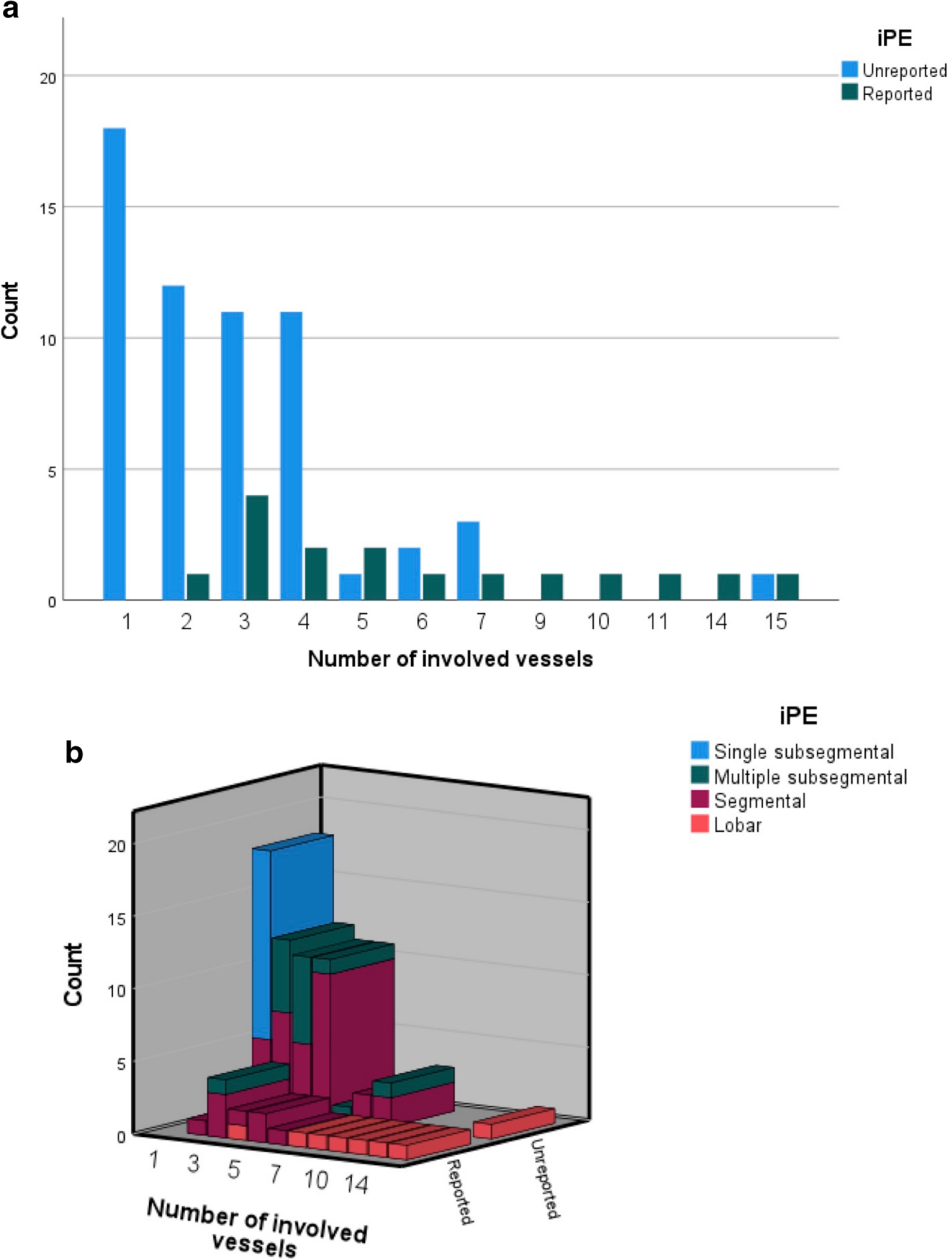
**a** Distribution of number of involved vessels, stratified for reported or unreported iPE. Unreported iPE had significantly lower median number of involved vessels, 2 vs 5 (*p* < 0.001). **b** Distribution of number of involved vessels, with the most proximal extent of the iPE shown

Per study, 16 studies had a reported iPE while 59 studies had an unreported iPE (total iPE prevalence 4.0%). Unreported iPE had a significantly lower number of involved vessels compared to reported iPE, with a median of 2 (IQR 1–4) versus 5 (IQR 3–9.75), *p* < 0.001 (Fig. 2a). Of the unreported iPE, 32/59 (54.2%) were proximal to the subsegmental arteries. In subsegmental and segmental iPE, the embolic burden varied between 1 and 7 involved vessels, and in lobar or more proximal iPE, between 4 and 15 vessels were involved (Fig. 2b). No significant differences were noted in age, cancer type, or enhancement of the main pulmonary artery between cases with unreported compared to reported iPE (281 vs 267 HU, *p* = 0.56).

Per patient, 53 patients were identified with an unreported iPE, 12 patients with a reported iPE, and 1004 patients without iPE. Baseline characteristics and extent of iPE are presented in Table 2, and stratified for unreported iPE, reported iPE, or no iPE. Compared to patients without iPE, patients with unreported iPE were more likely to have a cancer type with very high risk of VTE, having metastases, a central venous catheter (CVC), or being of female sex.

**Table 2:** Baseline characteristics of included patients. *BMI*, body mass index; *CVC*, central venous catheter; *iPE*, incidental pulmonary embolism; *IQR*, interquartile range; *NS*, not significant

	Without iPE	With unreported iPE	With reported iPE	
Total number of patients	1004	53	12	
Age, years	69.6 ± 11.6	70.6 ± 11.0	69.0 ± 8.0	NS
Women	48.7%	71.7%*	33.3%	*X*^2^ = 0.002
BMI	26.2 ± 6.3	24.6 ± 4.8	23.7 ± 3.3	NS
Cancer type				*X*^2^ < 0.001
Very high risk				
Pancreas	14	5	1	
Gastric	4	1	1	
Total	1.8%	11.3%*	16.6%*	
High risk				
Lymphoma	59	5	1	
Gynecologic	41	6		
Bladder	15	1		
Lung	133	8	3	
Testicular	9			
Total	25.6%	37.7%	33.3%	
Low risk				
Colorectal	207	10	3	
Esophageal	6	1		
Breast	142	9	1	
Prostate	96		2	
Hematologic	36			
Melanoma	39	3		
Renal	79	1		
Other	117	2		
Multiple	8	1		
Total	72.7%	50.9%*	50.0%	
Distant metastases	35.7%	61.7%*	72.7%*	*X*^2^ < 0.001
CVC/Port-a-cath	23.9%	50.9%*	58.3%*	*X*^2^ < 0.001
Extent and level of iPE				*X*^2^ < 0.001
Lobar		1 (1.9%)	7 (58.3%)**	
Segmental		30 (56.6%)	5 (41.7%)	
Subsegmental, multiple		13 (24.5%)		
Subsegmental, single		9 (17.0%)		
Median number of involved vessels (IQR)		3 (1–4)	6.5 (3.25–10.75)**	*p* < 0.001

*Significantly different compared to patients without iPE

**Significantly different compared to patients with unreported iPE

To further explore the higher prevalence of unreported iPE in women, post hoc analyses were performed. In the total population, there were no significant differences between men and women regarding severity of cancer types, metastases, age, or body mass index (BMI). Significantly more women had a CVC (31.5% vs 21.4%, *p* = 0.001) and women had a higher mean HU attenuation in the main pulmonary artery (249 vs 208 HU, *p* < 0.001), with significantly more studies being of good to excellent quality including the subsegmental arteries (74.0% vs 63.2%, *p* < 0.05). When comparing only patients with iPE, there were no significant differences in number of involved vessels between men and women (median 3 vs 3, *p* = 0.9).

The AI algorithm correctly identified 68 of 75 iPE with three false positives (sensitivity 90.7%, specificity 99.8%, PPV 95.6%, NPV 99.6%). False negatives occurred in cases with 1–3 involved vessels, none of which was originally reported. False positives were deemed to be caused by flow artifacts (*n* = 2) or perivascular infiltrates (*n* = 1). The AI compatibility analysis correctly identified 1888/1892 studies, whereas four studies were erroneously classified as NECT.

## Discussion

### Reported and unreported iPE

In the present study including patients with cancer in routine practice from a regional hospital, unreported iPE were common. While the total prevalence was 4.0%, only 21% were initially reported. Smaller iPE were more likely to be overlooked, but > 50% of patients had an unreported iPE proximal to the subsegmental arteries.

**Fig. 3:**
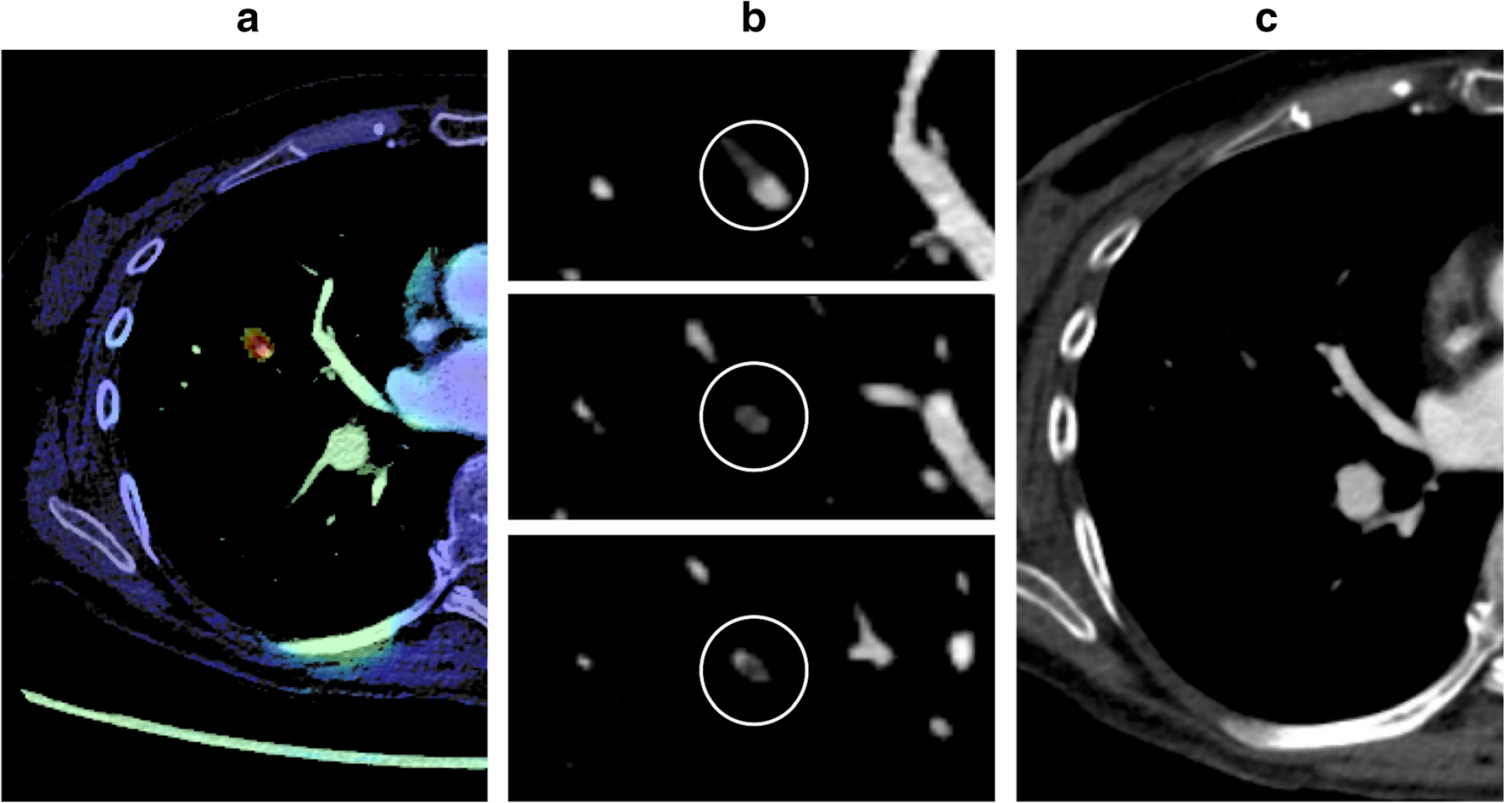
Unreported subsegmental iPE in a patient with ovarian cancer. **a** AI heatmap indicating a potential PE. **b** Intraluminal filling defect with typical features of a PE, visible on several images. **c** In the reconstructed 5-mm images, the area is only visible on a single image, showing no evidence of PE

**Fig. 4:**
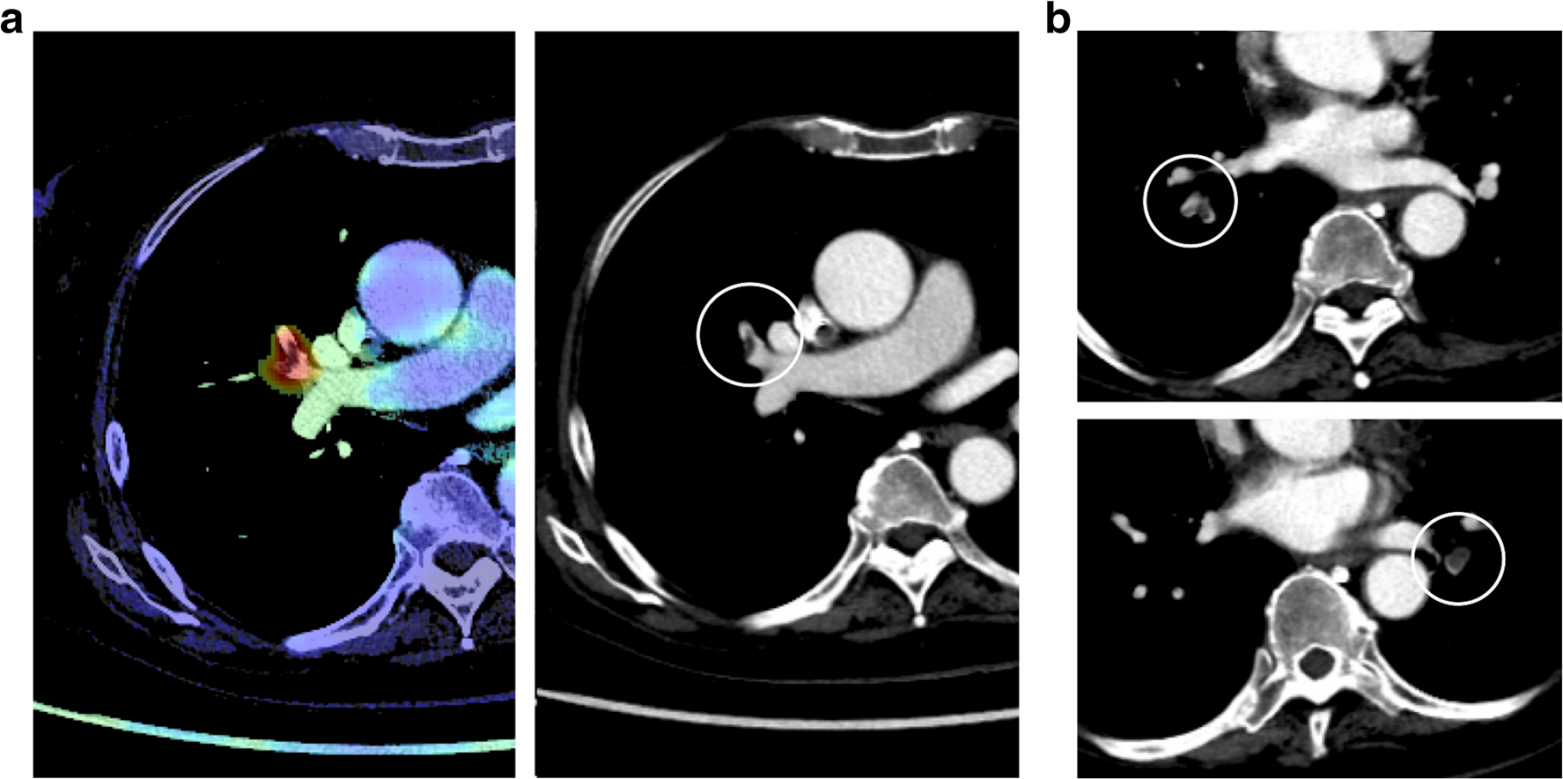
Unreported lobar and multiple segmental iPE in a patient with pancreatic cancer. **a** AI heatmap indicating a potential PE, clearly visible on the 5-mm images. **b** More caudally, there is evidence of additional multiple segmental PE in both lungs

The most likely reason for the underreporting of iPE is search error rather than interpretation error (Figs. 3 and 4). As iPE most often have a very typical appearance, it is unlikely that the reporting radiologist detected an iPE and then deemed it to be a false positive finding. Since pulmonary artery opacification was high and thin slices were always available, underreporting is likely related to an unsystematic review of the pulmonary arteries, and underutilization of the thin slices. Tresoldi et al showed that there was a significant improvement in sensitivity (from 46–50% to 82–92%) when CT slice thickness was decreased from 5 to 1.25 mm [14]; in the present study, the thin axial slices, together with thicker reconstructed slices, were always sent to the PACS. These results are similar to the study by Bach et al who showed a 3.9% prevalence of iPE in cancer patients, of which almost 60% were unreported [8].

The clinical impact of unreported iPE is less well known. Gladish et al showed that in cancer patients only 25% of iPE were reported in the initial interpretation, and that missed iPE tended to be smaller and isolated [5]. In their study, four out of 12 patients with missed iPE had a subsequent diagnosis of DVT while eight out of 12 had no evidence of recurrent VTE at follow-up, and resolution of the iPE was demonstrated at the first follow-up examination in all patients. Engelke et al evaluated the clinical outcome of patients with unreported PE and showed that while smaller PE were more likely to go undetected, patients with untreated iPE had no adverse 1-year survival compared to patients with treated iPE [6]. However, their material was heterogenous, including both patients with and without cancer, and different types of CT scans. In contrast, Sun et al showed that in patients with lung cancer with reported iPE, untreated iPE was associated with worse overall survival [2], and previous studies have shown a comparable recurrent VTE risk for subsegmental iPE compared to more proximal iPE [15, 16]. In addition, in a prospective management study on non-cancer patients with isolated subsegmental PE and without DVT who did not receive anticoagulant treatment, during the 90-day follow-up period the cumulative incidence of recurrent VTE was 3.1% (2.1% for a single subsegmental PE and 5.7% for multiple subsegmental PE), which was higher than anticipated [17].

In the present study, there were significantly more women than men with unreported iPE. Since the number of patients with unreported iPE was rather small, this could be a type I error. However, women were more likely to have a CVC, which could mean that the proportion of women receiving chemotherapy also was higher, thus reflecting a higher baseline risk of VTE in women in our material. As no data on chemotherapy was available, no firm conclusions can be made. In addition, in women more studies were of good to excellent quality to the subsegmental arteries, which could influence the prevalence of smaller iPE, as there were more opportunities to find subsegmental iPE in women. As contrast media volume in our study was determined by total body weight, it is likely that the higher proportion of good- to excellent-quality studies in women was due to women receiving on average a higher contrast media dose in relation to their circulating blood volume compared to men [18].

### Accuracy of the AI algorithm

**Fig. 5:**
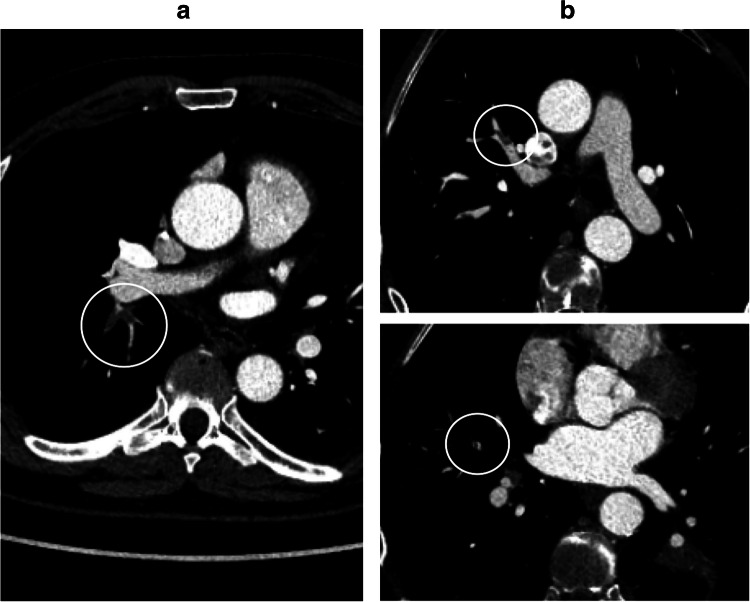
Unreported iPE detected at retrospective review, undetected by AI. **a** Segmental iPE in the apical segment of the right lower lobe, negative by AI. **b** Images from another patient with segmental and subsegmental iPE. The AI algorithm deemed this to be a NECT and the study was thus not further analyzed

The AI algorithm had a very high sensitivity and specificity with only three false positives and seven false negatives (Fig. 5). In our study, the significant underreporting of iPE in combination with the accuracy of the AI algorithm shows that there is potential for AI to increase the detection rate of iPE. In addition, the CT studies could be analyzed quickly by AI while the patients are still at the radiology department. Studies with suspicious findings could then be triaged to a radiologist to reduce report turnaround time, and potentially reduce time to treatment. The high accuracy of the AI algorithm, and the low number of false positives especially, is a prerequisite to be useful in the clinical workflow as each AI finding needs to be evaluated swiftly. However, several aspects need to be considered if such an AI algorithm is implemented in clinical practice: as there will be radiologists with varying experience in PE diagnosis using the AI algorithm, there is a potential risk of overdiagnosis due to false positive findings; in addition, there is a risk of unnecessary additional tests to confirm a smaller iPE, for example, with CTPA. A clearly defined workflow needs to be in place to reduce these risks.

### Limitations

There were several limitations to the present study. The prevalence of iPE could have been underestimated, as standard CT examinations are not optimized for opacification of the pulmonary arteries. In the present study ~30% of studies had either moderately reduced diagnostic confidence when evaluating the subsegmental arteries, or there was substantial uncertainty precluding evaluation of the subsegmental arteries. In addition, if a study was deemed to be negative after the first review and AI analysis, no additional read was performed. However, the prevalence of iPE was similar to previous retrospective studies.

### Summary

While the total prevalence of cancer-associated iPE was 4.0%, only 21% were reported. Smaller iPE were more likely to be overlooked, but greater than 50% of unreported iPE were proximal to the subsegmental arteries. As the AI algorithm had a very high sensitivity and specificity with very few false positives, it has the potential to increase the detection rate of iPE.

## Abbreviations

AI: Artificial intelligence
BMI: Body mass index
CECT: Contrast-enhanced CT
CTPA: CT pulmonary angiography
CVC: Central venous catheter
DVT: Deep venous thrombosis
iPE: Incidental pulmonary embolism
IQR: Interquartile range
NECT: Non-enhanced CT
PACS: Picture archiving and communication system
PE: Pulmonary embolism
VTE: Venous thromboembolism

## Declarations

### Guarantor

The scientific guarantor of this publication is Peder Wiklund.

### Statistics and biometry

No complex statistical methods were necessary for this paper.

### Methodology

• Retrospective

• Cross-sectional study

• Performed at one institution
